# Integration of Interleukin-6 Improves the Diagnostic Precision of Metagenomic Next-Generation Sequencing for Infection in Immunocompromised Children

**DOI:** 10.3389/fmicb.2022.819467

**Published:** 2022-03-08

**Authors:** Di Wang, Min Lai, Hua Song, Jing-Ying Zhang, Fen-Ying Zhao, Juan Liang, Wei-Qun Xu, Yong-Min Tang, Xiao-Jun Xu

**Affiliations:** ^1^Division/Center of Hematology-Oncology, The Children’s Hospital of Zhejiang University School of Medicine, Hangzhou, China; ^2^The Pediatric Leukemia Diagnostic and Therapeutic Technology Research Center of Zhejiang Province, National Clinical Research Center for Child Health, Hangzhou, China

**Keywords:** metagenomic next-generation sequencing, interleukin-6, pathogen, immunocompromised, children

## Abstract

The performance of metagenomic next-generation sequencing (mNGS) in identifying pathogens in immunocompromised children was not very clear. The purpose of this study is to assess the performance of mNGS in this population and to investigate whether the integration of serum cytokines and mNGS assay could improve diagnostic accuracy. We retrospectively collected the clinical data of pediatric patients who suffered febrile diseases and underwent mNGS determination simultaneously in the department of hematology/oncology between January 2019 and March 2021. Specimens were sent for conventional microbiological test (CMT), mNGS, and serum cytokine measurement in parallel. A total of 258 episodes of febrile diseases were enrolled, mNGS was positive in 224 cases, while CMT was positive in 78 cases. mNGS and CMT were both positive in 70 (27.1%) cases and were both negative in 26 (10.1%) cases. There were 154 (59.7%) cases positive by mNGS only while 8 (3.1%) were positive by CMT only. It was common that two or more pathogens were simultaneously detected by mNGS in a single specimen, with only 61 tests identified a single organism. Whether the organisms reported by mNGS were the microbiological etiology of infection was evaluated. Of the 224 cases with positive mNGS results, 135 (58.4%), 30 (13.0%), and 59 (28.6%) were considered as “probable,” “possible,” and “unlikely,” respectively. Patients with high IL-6 (≥ 390 pg/ml) were likely to be bacterial infection. Although mNGS reported mixed pathogens, 84.6% (33/39) and 83.3% (10/12) of patients presenting high IL-6 were confirmed as bacterial infection in the training and validation cohort, respectively. In conclusion, mNGS analysis demonstrates promising diagnostic potential in rapidly identifying clinically relevant pathogens. Given the detection of many clinically irrelevant organisms, the integration of IL-6 improves the precision of mNGS results interpretation.

## Introduction

Infection is one of the leading causes of mortality in children with hematology/oncology diseases and hematopoietic stem cell transplantation (HSCT) due to intensive chemotherapy and immunosuppression (Koc et al., 2013; Styczynski et al., 2016; Zhu et al., 2020). Early identification of pathogens is crucial for implementing effective pathogen-targeted therapies to prevent avoidable death (Kumar et al., 2006). However, it remains highly challenging to achieve the goal due to the limitations of culture-based methods in terms of sensitivity, speed, and spectrum of available assay targets. Moreover, the positivity of microbiologic culture is quite low in this population, especially for patients with febrile neutropenia (FN) (Xu et al., 2019). Other tests, such as microscopic examination, serology test and polymerase chain reaction (PCR) constrains the types and numbers of microorganisms that can be assessed by these methods.

Metagenomic next-generation sequencing (mNGS) is a high-throughput approach by which all genetic material in a biologic specimen is sequenced at a very high depth simultaneously (Besser et al., 2018; Chiu and Miller, 2019). In contrast to targeted methods, it enables the detection of nearly all known pathogens simultaneously from clinical specimens. Thus, mNGS is a useful technology to identify novel or unexpected pathogens (Hu et al., 2021). Previous studies showed that it is a very promising method to identify clinically relevant pathogens in immunocompromised patients (Horiba et al., 2018; Lee et al., 2020; Wang et al., 2020; Zhan et al., 2021), but the data on its performance in pediatric hematology/oncology patients was limited.

One pitfall of mNGS is the host genetic material in the specimens and the colonized microbial contamination creates noisy background, which makes it extremely challenging to differentiate true pathogen from colonization and opportunistic pathogens. Our previous studies have shown that inflammatory cytokines including IL-6, IL-10, TNF-α, and IFN-γ can be used for differentiating various pathogens. Patients infected with gram-positive and negative bacterial, fungal, and viral pathogens presented distinct cytokine profiles (Tang et al., 2011; Xu et al., 2013; Shen et al., 2016). In order to assess the ability of mNGS to detect pathogens in pediatric hematology/oncology patients and to investigate whether the integration of serum cytokines and mNGS assay could improve the sensitivity and accuracy of pathogen detection, we retrospectively collected and analyzed the data of patients who suffered febrile diseases and underwent mNGS determination in our department.

## Patients and Methods

### Study Subjects

We retrospectively collected the clinical data of pediatric patients who suffered febrile diseases and underwent mNGS determination simultaneously in the department of hematology/oncology at the Children’s Hospital of Zhejiang University School of Medicine between January 2019 and March 2021. Patients with febrile diseases who fulfilled the following criteria were enrolled for further analysis: (1) Patients were diagnosed with underlying diseases of leukemia, lymphoma or other malignancies, or diseases need HSCT, and they suffered from febrile diseases during chemotherapy or HSCT period. (2) Fever was defined as one ear temperature reading of > 38.5°C or at least two measurements of 38.0°C–38.4°C within a 24-h period. (3) Specimens were subjected to regular clinical microbiological assays and mNGS test in parallel. (4) The mNGS specimens should be taken from the infectious lesions. For example, for patients with bloodstream infection (BSI), the mNGS specimens should be peripheral blood; for patients with respiratory tract infection (RTI) and pneumonia, the mNGS specimen could be nasopharyngeal swab (NPS), sputum, or bronchoalveolar lavage fluid (BALF); for patients with multiple lesion infection, specimens from either lesion could be used for evaluation. Patients without a clear infection site or positive microbiological result by conventional microbiological test (CMT) were assigned into fever of unknown origin (FUO) group. For patients with FUO, blood specimen was used for evaluation. This study was conducted in accordance with the Declaration of Helsinki. Study protocols were reviewed and approved by the Children’s Hospital of Zhejiang University School of Medicine Institutional Review Board (IRB) and Ethics Committee (IRB number 2021-IRB-004).

### Study Definition

CMT was defined as bacterial and fungal culture; multiplex PCR for common respiratory virus (respiratory syncytial virus, influenza virus, parainfluenza virus, and adenovirus), human herpes virus, cytomegalovirus (CMV), Epstein–Barr virus (EBV), and *M. pneumoniae*; serum 1,3 β-D-glucan for fungal infection; IgM and IgG antibody test for human herpesvirus and *Aspergillus fumigatus*; and T-spot assay for tuberculosis. For microbiological culture, if the culture showed bacterial or fungal growth, strain identification was conducted by Matrix-Assisted Laser Desorption/Ionization Time of Flight Mass Spectrometry (MALDI-TOF MS, Bruker, Germany). For patients with positive CMT results, the microorganisms consistent with CMT results were considered as the pathogens. For patients with negative CMT results, in order to determine if the reported organisms by mNGS were the microbiological etiology of febrile diseases, cases were reviewed individually by two investigators (D Wang and M Lai) independently to evaluate the possibility based on the following items and make a preliminary determination, which was then adjudicated by Dr. XJ Xu if necessary: (1) the inherent pathogenicity of the organism and the potential for the organism to cause infectious diseases in immunocompromised children; (2) whether the clinical history and feature was likely caused by the organism, i.e., pulmonary lesion was common in aspergillosis infection while *Candida* species were not considered as pathogens of pneumonia. CMV was considered as etiology only in patients who underwent HSCT or very intensive chemotherapy; and (3) whether empirical antimicrobial therapy before mNGS likely had activity against the organisms and whether the organisms responded to the antimicrobial regimens administered according to the mNGS results. The results were divided into the following three situations:

(1) **Probable:** The organism was deemed to be pathogenic, consistent with the clinical characteristics and not covered by previous antimicrobial therapy but cured after the adjustment of antimicrobial regimens based on mNGS results.
(2) **Possible:** The organism was deemed to be pathogenic, consistent with the clinical characteristics, but the organisms were likely covered by the previous antimicrobial regimens or non-response to the adjustment of antimicrobial therapy based on mNGS results.
(3) **Unlikely:** The detected organisms were non-pathogenic, did not fit the clinical features, and/or did not require treatment or non-response to the antimicrobial therapy based on mNGS results.

### Metagenomic Next-Generation Sequencing Analysis

Different samples were collected from patients according to standard procedures for mNGS analysis, which were immediately transported on dry ice to a commercial laboratory for mNGS testing within 8 h after collection. The process of mNGS consisted of library preparation, metagenomic sequencing, and bioinformatics pipeline analysis. DNA library was constructed through sample processing, nucleic acid extraction, enzymatic fragmentation, end repair, terminal adenylation, and adaptor ligation (Luan et al., 2021). The quality of DNA libraries was checked by real-time quantitative PCR (qPCR) using a KAPA Library Quant Kit (Illumina) Universal qPCR Mix (KAPA Biosystems Pty). Each well added 6 μl of qPCR Premix and 4 μl of sample or standard in PCR plates. The concentrations of the standards were 20, 2.0, 0.2, 0.02, and 0.002 pM. After mixing and centrifuging, the PCR reactions were performed in a Gentier 96R real-time qPCR system machine (Tianlong, Xi’an, China). Finally, the sample quality was calculated using the following formula: the quality of DNA library = qPCR count × the fragment size of standard/the average fragment size of the DNA library. Shotgun sequencing (50 bp, single-end) was performed on the Illumina Nextseq platform. Culture medium containing 10^4^ or 10^6^ Jurkat cells/ml served as negative control. Each library generated about 20 million reads. Low-quality and short reads were removed and human origin sequences aligned to the human reference genome (GRCh38.p13) were filtered. Afterward, the remaining reads were compared with the microbial reference database to determine the microbial species. The microbial reference database included NCBI nt, GenBank, and in-house curated genomic database, which contained 11,162 bacterial genomes or scaffolds, 11,704 whole-genome sequences of viral taxa, 1,324 fungi, and 229 parasites. The number of alignment reads and abundance was calculated.

Microbial reads identified from a library were reported if they met following criteria: (1) the sequencing data passed quality control filters (library concentration > 50 pM, Q20 > 85%, Q30 > 80%); (2) the species did not contain in the negative control of the same sequencing run or the ratio of reads per million total reads (RPM) of sample to RPM of negative control ≥ 5, an empirical cutoff for discriminating true-positives from background contaminations according to previous studies (Schlaberg et al., 2017; Wilson et al., 2019; Luan et al., 2021).

### Cytokine Analysis

The blood samples for cytokine determination were collected simultaneously with the blood sample for mNGS, or within 24 h before or after specimens from other sites were taken for mNGS analysis. The blood specimen was transferred to a serum-separating tube and the serum was harvested for analysis. The concentrations of IFN-γ, TNF-α, IL-10, IL-6, IL-4, and IL-2 were quantitatively determined by the CBA human Th1/Th2 cytokine kit II (BD Biosciences, San Jose, CA, United States) as described previously (Tang et al., 2008). The minimal and maximum limits of detection for all six cytokines were 1.0 pg/ml and 5,000.0 pg/ml, respectively. For values higher than 5,000 pg/ml, 5,000 pg/ml was used in the statistical analysis.

### Statistical Analysis

Comparative analyses were conducted by using the chi-square test, Fisher’s exact test for categorical variables, and Mann–Whitney *U* test for continuous variables. The performance of inflammatory biomarkers to predict bacterial infection was assessed and compared with receiver operating characteristics (ROC) curves, and the area under the curve (AUC) and optimal cutoff values were calculated. All statistical analyses were performed using SPSS version 20.0 (IBM, Armonk, NY, United States). *p* < 0.05 was considered to be statistically significant.

## Results

### Patients’ Characteristics

**FIGURE 1:**
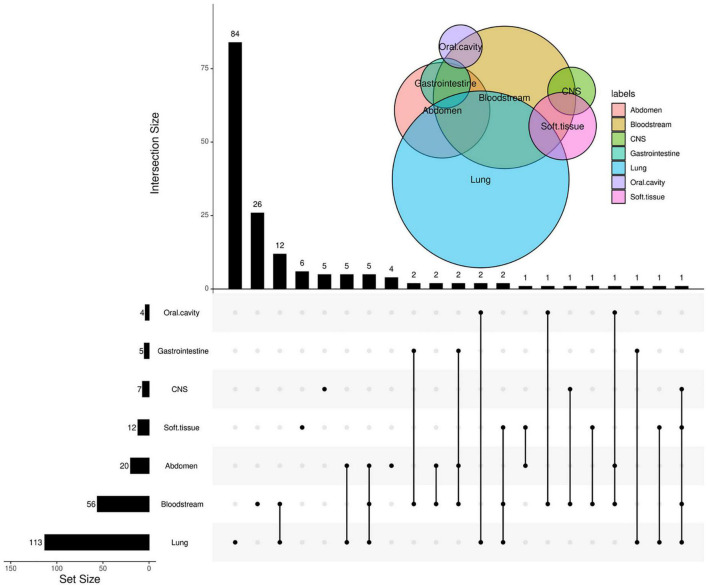
Venn Upset plot of the distribution of sites of infection. The Venn diagram in the right upper area represents the intersection for different sites of infection. In the upset diagram, each row represents the site of infection; the number at the end of each bar represents the total cases with this site infection, and each column represents the case number of every situation, especially patients with multiple sites of infection. The number at the end of each bar represents the cases with infection at the sites the “black dots” located. The black dots connected by line indicate multiple infections.

In the present study, a total of 258 episodes of febrile diseases were enrolled. As to the types of infection, 113 were diagnosed with RTI or pneumonia, 92 with FUO, 56 with BSI, 20 with abdominal infection, 12 with soft tissue infection, 7 with central nervous system infection, 5 with gastrointestinal infection, and 4 with oral cavity infections. Forty patients presented more than one lesion infection (Figure 1). The median age of the patients was 5.4 years old (range, 0.1–16.3), with the male-to-female ratio of 1.36:1. The main underlying diseases or conditions of these patients were acute lymphoblastic leukemia (*n* = 152), acute myeloid leukemia (*n* = 37), and lymphoma (*n* = 17), HSCT (*n* = 26). As to the neutrophil count, 194 (75.2%) patients underwent FN, while 64 patients’ absolute neutrophil count (ANC) was more than 0.5 × 10^9^/L. The median interval from the onset of febrile disease to the mNGS specimen taken was 5 days.

### Distribution of Microorganisms

**FIGURE 2:**
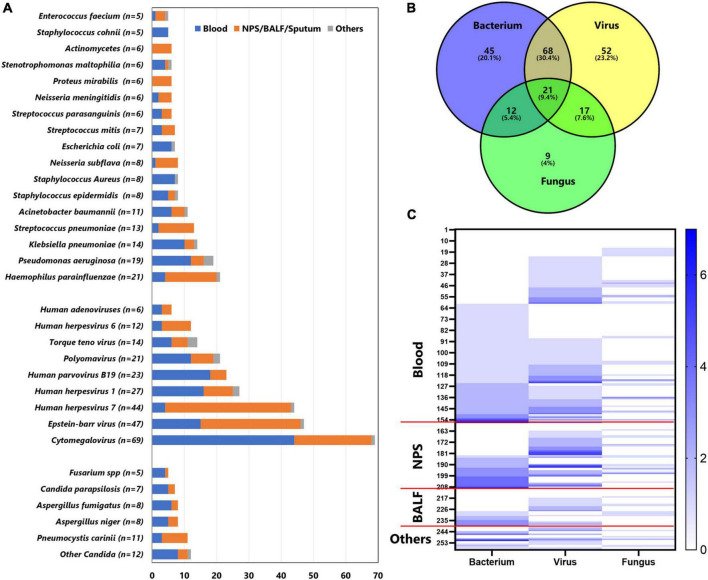
Distribution of main pathogens identified in this cohort by mNGS. **(A)** The figure showed the number of organisms detected in different specimens. Blue bars indicate organisms detected in blood, orange bars indicate organisms detected in nasopharyngeal swab (NPS), bronchoalveolar lavage fluid (BALF), or sputum; gray bars indicate organisms detected in other specimens (pus, hydrothorax, ascites, and cerebrospinal fluid). **(B)** Venn diagram represents the intersection for different microbiological species. **(C)** Heatmap represents the number of bacterium, virus, and fungus detected in each specimen. The left *y*-axis indicates the case number. The counts of bacterium, virus, and fungus in the same specimen are shown in color, with larger counts associated with darker colorings.

Overall, mNGS was positive in 224 tests, identifying 115 strains of bacteria, 38 fungi, and 22 viruses, while CMT was positive in 78 tests, identifying 21 bacteria, 4 fungi, and 7 viruses. The main organisms detected by mNGS are shown in Figure 2A: *Haemophilus parainfluenzae, Pseudomonas aeruginosa, Klebsiella pneumoniae, Streptococcus pneumoniae*, and *Acinetobacter baumannii* were the most common bacteria; *Cytomegalovirus, Epstein–Barr virus, human herpesvirus 7, human herpesvirus 1*, and *human parvovirus B19* were the most common viruses; and *Pneumocystis carinii, Aspergillus niger, Aspergillus fumigatus, Candida parapsilosis*, and *Fusarium spp.* were the most common fungi, while the most frequent organisms detected by CMT were *coagulase-negative Staphylococcus* (*n* = 12), *Pseudomonas aeruginosa* (*n* = 9), *Klebsiella pneumoniae* (*n* = 6), *Escherichia coli* (*n* = 4), *Staphylococcus aureus* (*n* = 4), *Stenotrophomonas maltophilia* (*n* = 4), *Cytomegalovirus* (*n* = 5), *Epstein–Barr virus* (*n* = 5), and *parainfluenza virus* (*n* = 6). It is common that two or more pathogens were simultaneously detected by mNGS in a single specimen, with only 61 tests identifying a single organism. Of the 224 tests, mNGS reported one type of organism (bacterium, virus, or fungus) in 106 tests, two types of organisms in 97 tests, and three types of organisms in 21 tests (Figure 2B). The “co-infection” of bacterium and virus according to mNGS accounted for 29.4% of positive tests. Moreover, in tests with mNGS-proven bacteria, 48.3% presented two or more strains of bacteria; similarly, 50.9% of tests with mNGS-proven virus and 20.0% of tests with mNGS-proven fungi reported two or more strains of viruses or fungi, respectively (Figure 2C).

### Diagnostic Performance Comparison of Metagenomic Next-Generation Sequencing and Conventional Microbiological Test

**FIGURE 3:**
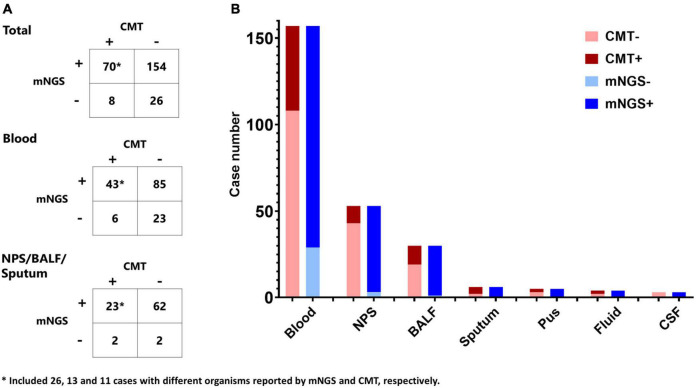
Comparison of mNGS and conventional microbiological tests (CMT). **(A)** Contingency tables for the agreement of mNGS and CMT in total cohort, blood specimen, and nasopharyngeal swab (NPS)/bronchoalveolar lavage fluid (BALF)/sputum. **(B)** Comparison of the positive cases of mNGS and CMT in different specimens.

Of the 258 specimens, 157 were blood, 53 were NPS, 30 were BALF, 6 were sputum, 5 were pus, 4 were hydrothorax and ascites, and 3 were cerebrospinal fluid. Metagenomic NGS and CMT were both positive in 70 (27.1%) cases and were both negative in 26 (10.1%) cases. Of the 70 cases, mNGS and CMT had agreement in 44 cases, and they reported different organisms in another 26 cases. There were 154 (59.7%) cases positive by mNGS only while 8 (3.1%) were positive by CMT only (Figure 3A). As to different types of specimens, the positive rates of blood, NPS, and BALF were 81.5, 94.3, and 96.7% for mGNS and 31.2, 18.9, and 36.7% for CMT (Figure 3B). Compared with blood, specimens from respiratory tract and lung presented much lower agreement between mNGS and CMT (53/157 vs. 14/89, *p* = 0.002). Of 92 patients diagnosed as FUO according to CMT, mNGS results were positive in 75 cases, including 28 “probable” cases, 11 “possible” cases, and 36 “unlikely” cases.

### Assessment of the Metagenomic Next-Generation Sequencing Results to Be Microbiological Etiology

Based on CMT and mNGS, 182 patients were diagnosed with microbiology etiology, including 89 cases of bacterial infection, 44 cases of viral infection, 35 cases of fungal infection, 9 cases of other pathogen infection, and 5 cases of polymicrobial infection. Compared with CMT, mNGS provided additional detected organisms in many cases. As mNGS often reported several organisms in one specimen, whether the reported organisms by mNGS were the microbiological etiology of febrile diseases was further investigated based on the pathogenicity of the organism, clinical characteristics, infection lesions, and treatment response. Of the 224 cases with positive mNGS results, 135 cases (60.3%) were considered as “probable,” 30 (13.4%) as “possible,” and 59 (26.3%) as “unlikely.” The detail of different types of specimens is shown in Table 1. The “probable” rates were 45.2% in blood specimens, 52.8% in NPS specimens, and 66.7% in BALF specimens, respectively. As to organism species, mNGS-reported virus presented a much lower “probable” rate compared with bacterium and fungus.

**TABLE 1:** The probability of mNGS-detected organisms to be etiology of infection in different specimens.

	Specimen						Organism				
	Blood (*n* = 157)	NPS (*n* = 53)	BALF (*n* = 30)	Sputum (*n* = 6)	Others (*n* = 12)	*p*-value*	Bacterium (*n* = 147)	Virus (*n* = 163)	Fungus (*n* = 60)	Others (*n* = 23)	*p*-value**
Probable	71 (45.2%)	28 (52.8%)	20 (66.7%)	5 (83.3%)	11 (91.7%)	0.031	70 (47.6%)	27 (16.6%)	32 (53.3%)	6 (26.1%)	< 0.001
Possible	18 (11.5%)	6 (11.3%)	5 (16.7%)	0 (0%)	1 (8.3%)	0.834	11 (7.4%)	13 (8.0%)	6 (10.0%)	1 (4.3%)	0.831
Unlikely	38 (24.2%)	16 (30.2%)	4 (13.3%)	1 (16.7%)	0 (0%)	0.914	66 (44.9%)	123 (75.5%)	22 (36.7%)	16 (69.6%)	< 0.001
Negative	30 (19.1%)	3 (5.7%)	1 (3.3%)	0 (0%)	0 (0%)	0.001					

*Comparison between blood and respiratory samples (NPS + BALF + sputum).

**Comparison among bacterium, virus and fungus.

### Neutropenia and Metagenomic Next-Generation Sequencing

To further investigate the impact of neutropenia on the results of mNGS, we compared the potential pathogens between patients with absolute neutrophil count (ANC) < 0.5 × 10^9^/L and those with ANC ≥ 0.5 × 10^9^/L. The positive rates of CMT were comparable between patients with and without FN (63/194 vs. 15/64, *p* = 0.172), while the positive rate was much higher in patients with ANC ≥ 0.5 × 10^9^/L (163/194 vs. 61/64, *p* = 0.021). Neutropenia presented no impact on the agreement of mNGS and CMT (28.9% vs. 20.3%, *p* = 0.051). However, in neutropenic patients, the mNGS-reported organisms were much less “probable” to be the etiology of infection when compared with those with ANC ≥ 0.5 × 10^9^/L (45.9% vs. 71.9%, *p* = 0.001). As to the different organisms, virus detected by mNGS presented a lower rate of true positive in neutropenic patients (20/124 vs. 20/39, *p* < 0.001), while bacterium (59/111 vs. 21/36, *p* = 0.588) and fungus (27/47 vs. 9/13, *p* = 0.443) were comparable between neutropenic patients and those with ANC ≥ 0.5 × 10^9^/L.

### Inflammatory Biomarkers and Metagenomic Next-Generation Sequencing

**FIGURE 4:**
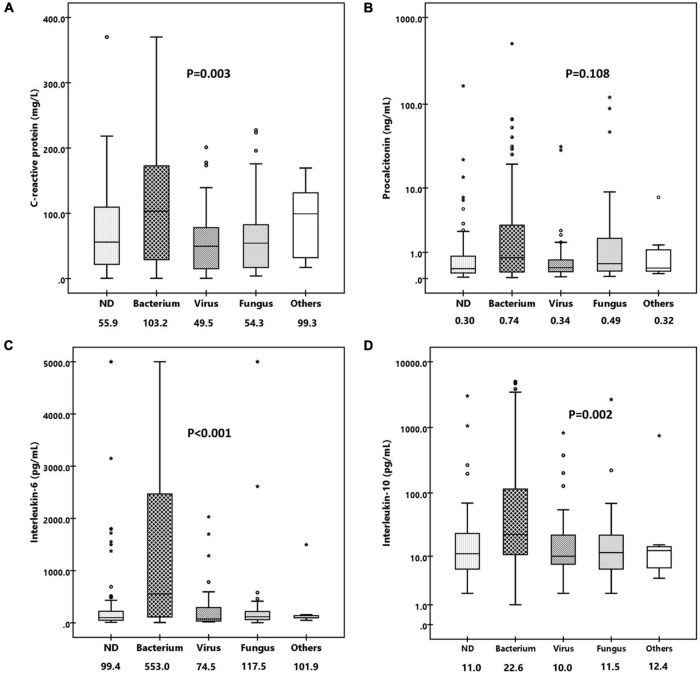
Comparison of levels of various inflammatory biomarkers in patients infected with bacterium, virus, fungus, and others. **(A)** Box-and-whisker plots of C-reactive protein. **(B)** Box-and-whisker plots of procalcitonin. **(C)** Box-and-whisker plots of IL-6. **(D)** Box-and-whisker plots of IL-10. Median levels (lines), 25th–75th percentiles (box), and 5th–95th percentiles (whiskers) are shown for each biomarker. The values of median concentrations are shown in the figures.

**FIGURE 5:**
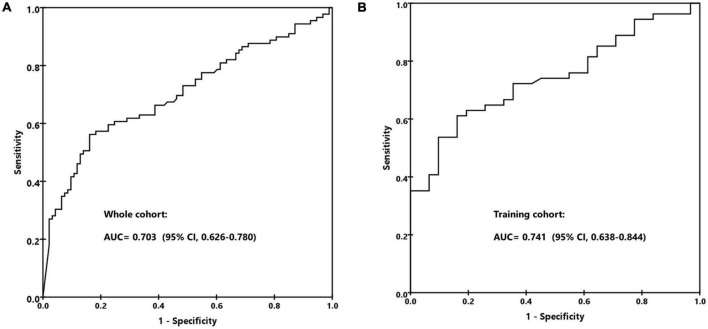
The receiver operating characteristic (ROC) curve for predicting bacterial infection by IL-6. **(A)** The whole cohort. **(B)** The training cohort.

As the inflammatory biomarkers can be used for the differentiation of various organisms, we investigate the performance of C-reactive protein (CRP), procalcitonin (PCT), IL-6, IL-10, TNF-α, and IFN-γ to distinguish infections caused by different pathogens. As shown in Figure 4, patients with confirmed bacterial infection presented much higher levels of CRP (median, 103.2 mg/L vs. 49.5 mg/L vs. 54.3, *p* = 0.003), IL-6 (median, 553.0 pg/ml vs. 74.5 pg/ml vs. 117.5 pg/mL, *p* < 0.001), and IL-10 (median, 22.6 pg/ml vs. 10.0 pg/ml vs. 11.5 pg/ml, *p* = 0.002) than those with viral or fungal infection, while PCT (median, 0.74 ng/ml vs. 0.34 ng/ml vs. 0.49 ng/ml, *p* = 0.108), TNF-α (median, 2.0 pg/ml vs. 1.3 pg/ml vs. 1.7pg/ml, *p* = 0.057), and IFN-γ (median, 5.9 pg/ml vs. 6.5 pg/ml vs. 4.7 pg/ml, *p* = 0.423) levels were comparable. We then tested the performance of CRP, IL-6, and IL-10 to predict bacterial infection; the AUCs of CRP, IL-6, and IL-10 were 0.614 (95% CI, 0.531–0.697), 0.703 (95% CI, 0.626–0.780) (Figure 5A), and 0.657 (95% CI, 0.577–0.737), indicating IL-6 was the most powerful biomarker to predict bacterial infection.

**FIGURE 6:**
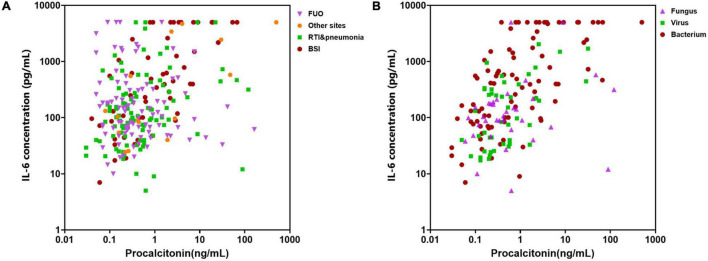
Distribution of cases with different infectious sites or pathogens based on procalcitonin (PCT) and interleukin (IL)-6 levels. **(A)** Distribution of cases with different infectious sites. FUO, fever of unknown origin; RTI, respiratory tract infection; BSI, bloodstream infection. **(B)** Distribution of cases caused by different microorganisms.

The above biomarkers’ levels in different infectious sites were compared as well, which were divided into BSI, RTI and pneumonia, FUO, and others. The PCT and IL-6 levels in patients with BSI were much higher than other sites, while the levels of CRP and IL-10 did not present a very significant difference in quantity although the p values were less than 0.05 (BSI vs. RTI & pneumonia vs. FUO vs. others, median levels: CRP, 87.2 mg/L vs.52.6 mg/L vs. 74.7 mg/L vs. 47.2 mg/L, *p* = 0.002; PCT, 0.89 ng/ml vs. 0.38 ng/ml vs. 0.43 ng/ml vs. 0.27 ng/ml, *p* = 0.002; IL-6, 397.3 mg/L vs. 107.9 mg/L vs.117.9 mg/L vs. 128.5 mg/L, *p* = 0.003; IL-10, 25.1 pg/ml vs. 12.0 pg/ml vs. 15.4 pg/ml vs. 10.7 pg/ml, *p* < 0.001). We thus investigated the distribution of patients according to their infectious sites or pathogens based on PCT and IL-6. The results showed that these two markers together performed well to identify patients with bacterial infection, while they failed to distinguish different infectious sites (Figure 6).

### Integrating IL-6 and Metagenomic Next-Generation Sequencing to Improve Diagnostic Accuracy

We randomly split the patients with confirmed organisms (*n* = 182) into training cohort and validation cohort according to a ratio of 2:1. The gender, age, neutropenia, specimen distribution, CMT, and mNGS positivity; CRP, PCT, IL-6, and IL-10 levels; and the agreement rate of mNGS with clinic were all comparable between the two groups (Table 2). In the training cohort, the AUC of IL-6 to predict bacterial infection was 0.741 (95% CI, 0.638–0.844), with a specificity of 85% and sensitivity of 61% at the cut-off value of 390 pg/ml (Figure 5B). We divided the patients into low (< 390 pg/ml) and high (≥ 390 pg/ml) IL-6 groups, which indicated unlikely and likely bacterial infection. The bacterial infection accounted for 35.0% (27/77) of patients with low IL-6 and 77.3% (34/44) of patients with high IL-6 (*p* < 0.001). The positive likelihood ratio (+ LR), negative likelihood ratio (-LR), and diagnostic odds ratio were 3.30, 0.54, and 6.11, respectively. Of 85 patients with mNGS-detected bacteria, bacterium, virus, and fungus were all reported in 14 cases, bacterium and virus in 34 cases, bacterium and fungus in 9 cases, and only bacterium in 28 cases. Of the above patients with mixed pathogens, 84.6% (33/39) of cases presenting high IL-6 were finally confirmed as bacterial infection.

**TABLE 2:** Comparison of features of training set and validation set.

Parameters	Training set (*n* = 121)	Validation set (*n* = 61)	*P* value
Male	73 (60.3%)	42 (68.9%)	0.260
Age (median and range)	4.5 (0.1-16.1)	6.0 (0.3-14.1)	0.762
Neutropenia	82 (67.7%)	43 (70.5%)	0.708
Specimen distribution			0.657
Blood	71 (58.7%)	32 (52.5%)	
NPS/BALF/Sputum	44 (36.4%)	23 (37.7%)	
CMT positivity	55 (45.5%)	21 (34.4%)	0.154
mNGS positivity	114 (94.2%)	60 (98.4%)	0.198
Confirmed bacterial infection	60 (49.6%)	33 (54.1%)	0.565
Probable and possible	92 (76.0%)	43 (70.5%)	0.420
CRP (mg/L)	73.0 (0.5-370)	53.3 (0.5-325.1)	0.452
PCT (ng/mL)	0.44 (0.03-120.0)	0.58 (0.05-499.1)	0.592
IL-6 (pg/mL)	171.0 (5.0-5000)	180.8 (14.5-5000)	0.574
IL-10 (pg/mL)	14.7 (2.0-5000)	12.4 (1.0-2672.0)	0.672

The validation cohort contained 61 cases, with 22 cases presenting IL-6 higher than 390 pg/ml. The AUC of IL-6 to predict bacterial infection was 0.679 (95% CI, 0.539–0.818). Of the 39 cases with low IL-6, 69.2% was confirmed as non-bacterial infection, while 72.7% of cases with high IL-6 were confirmed as bacterial infection. Of 25 patients that presented both bacterium and other pathogen in the mNGS results, 10 out of 12 (83.3%) patients with high IL-6 were finally confirmed as bacterial infection.

## Discussion

Precise and prompt microbiological diagnosis of febrile diseases is essential for the effective pathogen-targeted therapies. The results of the present study showed that mNGS is a sensitive technology to identify pathogens in pediatric hematology/oncology patients with febrile diseases. Compared with CMT, it presented a very high positivity rate, and many cases with negative CMT results attained microbiological diagnosis based on mNGS reports. However, as mNGS often reported several organisms in one specimen due to the colonized microbial contamination, the interpretation of the results is a big challenge. Serum cytokine IL-6 is a useful biomarker to differentiate bacterial and other pathogen infection, which can be integrated with mNGS to improve its diagnostic ability for the microbiological etiology of febrile diseases.

Compared with immunocompetent population, pediatric hematology/oncology patients present distinct features when suffering febrile diseases, which made it difficult for clinicians to interpret the mNGS results. Firstly, due to previous intensive chemotherapy and immunosuppressive agents, most patients are in neutropenic or immunocompromised status when they suffer febrile diseases. In this circumstance, polymicrobial infections are common (Rolston et al., 2007). In this cohort, mNGS reported two or more types of organisms in a single specimen in 51.2% cases, which makes it very different to judge what the true etiologic pathogen is.

Secondly, viruses were frequently detected in hematology/oncology patients with febrile neutropenia. Although usually shedding asymptomatically, they may lead to organ disease when they become opportunistic pathogens. Thus, it is a great challenge for clinicians to judge the causative role of a certain virus as mNGS is unable to differentiate colonization and pathogenic strains. As shown in the present study, viruses were not the true etiology of fever although they were reported by the mNGS in 75.5% of cases.

Thirdly, rare or atypical pathogens in immunocompetent patients were often reported by mNGS in this population. Organisms such as *Pneumocystis jirovecii, Aspergillus*, and *Candida*, which are difficult to identify using CMT, are often reported by mNGS (Tsang et al., 2021). Thus, it is a great challenge for clinicians to judge the causative role of certain pathogens as mNGS is unable to differentiate colonization and pathogenic strains (Geng et al., 2021).

Due to the above complicated situations, although mNGS can rapidly and accurately identify pathogens, its real-world clinical impact remains controversial. Some single-center studies from the children’s hospitals showed that this technology has good clinical performance with high sensitivity and provides clinically relevant information in most positive cases (Rossoff et al., 2019), while other studies found that mNGS had limited value for patient care. Recently, a multicenter retrospective cohort study of 82 plasma tests reported a positivity rate of 61%, but a positive clinical impact was identified in only 7.3% of cases (Hogan et al., 2021). A similar finding was reported in another study on mNGS on cerebrospinal fluid (Wilson et al., 2019). Given the detection of many clinically irrelevant organisms, the application of mNGS in routine clinical practice requires a more cautious interpretation of the results (Lee et al., 2020).

Thus, in order to improve the precision of diagnosis based on mNGS, we plan to integrate the inflammatory biomarkers into the mNGS results, facilitating to differentiate bacterial and non-bacterial infection. IL-6 is an important biomarker elevated during sepsis. As shown in our previous studies, together with IL-6, IL-10, and IFN-γ, the cytokine patterns are helpful in discriminating bacterial and virus infection, Gram-positive and negative bacterial infection, and invasive pulmonary aspergillosis and pneumocystis pneumonia (Tang et al., 2011; Xu et al., 2013; Shen et al., 2016). In the present study, the IL-6 level was much higher in patients confirmed as bacterial infection compared with those with non-bacterial infection. It performed much better to help to diagnose bacterial infection than other inflammatory biomarkers such as CRP, PCT, and IL-10. It was common that the mNGS specimens from patients who underwent bacterial infection were contaminated with virus or other organisms. For example, CMV and EBV were detectable in 69 (26.7%) and 47 (18.2%) cases but were documented as etiology of infection in only eight and six cases, respectively. Thus, besides mNGS results, the accurate microbiological diagnosis should be based on the clinical manifestation, laboratory findings and antimicrobial agents as well. After all, the reported organisms were confirmed as etiology of infection in about only 60-70% of cases with positive results. For patients with EBV infection, besides IL-6 and IL-10, IFN-γ usually elevated, which is different from bacterial infection. Lung, gut, and eye are the common target organs in CMV infection in patients who underwent HSCT. Cytokines are often within normal range or only slightly elevated as fever is not very common during CMV infection. However, when the patient underwent mixed pathogens co-infection, the pathogens which cause slight inflammation response may be ignored according to the cytokine results.

Our research also has certain limitations. Firstly, microbiological culture was regularly done before antibiotics administration, but almost all patients were pre-treated with antibiotics prior to mNGS. This may lead to the disparity of the two methods. Secondly, as this is a retrospective study, the time point for mNGS was not previously uniformly planned, and the positivity of mNGS may be affected. Thirdly, for pediatric hematology/oncology patients, it is not easy to ascertain whether the febrile disease is caused by infection for every case, especially those with neutropenic fever. This may influence the diagnostic efficiency evaluation of mNGS. To make the evaluation more objective, we split patients without a clear infection site or positive microbiological result into the FUO group and classified the mNGS results into probable, possible, and unlikely groups. Finally, as the mNGS determination is very expensive, not all patients with fever choose to do this. There is a selection bias in this study. Recently, we have launched a prospective study, in which patients with febrile neutropenia respond poorly to carbapenem and vancomycin/linezolid is enrolled for mNGS analysis. We believe this prospective study will add more information in this field.

## Data Availability Statement

The original contributions presented in the study are publicly available. This data can be found here: https://www.ncbi.nlm.nih.gov/sra/?term=PRJNA794318.

## Ethics Statement

The studies involving human participants were reviewed and approved by The Children’s Hospital of Zhejiang University School of Medicine Institutional Review Board and Ethics Committee. Written informed consent to participate in this study was provided by the participants’ legal guardian/next of kin.

## Author Contributions

DW, HS, J-YZ, F-YZ, JL, W-QX, Y-MT, and X-JX were the responsible pediatricians and participated in data collection. DW, ML, and X-JX performed data analysis. Y-MT and X-JX designed the work and performed data interpretation. DW and ML drafted the manuscript. X-JX revised the manuscript. All authors reviewed the manuscript and provided final approval.

## Conflict of Interest

The authors declare that the research was conducted in the absence of any commercial or financial relationships that could be construed as a potential conflict of interest. The reviewer HZ declared a shared affiliation with the authors to the handling editor at time of review.

## Publisher’s Note

All claims expressed in this article are solely those of the authors and do not necessarily represent those of their affiliated organizations, or those of the publisher, the editors and the reviewers. Any product that may be evaluated in this article, or claim that may be made by its manufacturer, is not guaranteed or endorsed by the publisher.
